# Ginsenoside Compound K Assisted G-Quadruplex Folding and Regulated G-Quadruplex-Containing Transcription

**DOI:** 10.3390/molecules26237339

**Published:** 2021-12-03

**Authors:** Yan Zhang, Zhidong Qiu, Ming Zhu, Ye Teng

**Affiliations:** School of Pharmacy, Changchun University of Chinese Medicine, 1035 Boshuo Road, Changchun 130117, China; zhangyan20201010@163.com (Y.Z.); qzd_ccucm@163.com (Z.Q.); zhuming124@163.com (M.Z.)

**Keywords:** ginsenoside CK, G-quadruplex, transcription

## Abstract

Ginsenoside compound K (CK) is one of the major metabolites of the bioactive ingredients in *Panax ginseng*, which presents excellent bioactivity and regulates the expression of important proteins. In this work, the effects of CK on G-quadruplexes (G4s) were quantitatively analyzed in the presence and absence of their complementary sequences. CK was demonstrated to facilitate the formation of G4s, and increase the quantity of G4s in the competition with duplex. Thermodynamic experiments suggested that the electrostatic interactions were important for G4 stabilization by CK. CK was further found to regulate the transcription of G4-containing templates, reduce full-length transcripts, and decrease the transcription efficiency. Our results provide new evidence for the pharmacological study of ginsenosides at the gene level.

## 1. Introduction

*Panax ginseng* is a natural herb that widely used in Chinese traditional medicine [1]. Many active ingredients extracted from ginseng, such as ginsenosides and polysaccharides, have been demonstrated to have pharmacological effects on cancer, diabetes, immune disorders, and neurodegeneration [2,3,4,5]. Ginsenosides in particular are considered as the most important ingredients, which have approximately 150 different structures [6]. Ginsenoside compound K (CK) is the major metabolite of protopanaxadiol ginsenosides, and shows excellent bioactivity in inhibiting the activity and proliferation of cancer cells and inducing apoptosis [7]. For example, Zhang reported that CK induced HepG2 cell cycle arrest, blocked cell cycle progression, and induced apoptosis [8]. CK was also found to enhance apoptosis by the downregulation of Akt-1 in SKBR3 cells [9]. However, most related research has focused on the effects of CK on protein expression level, and less information has been reported about the interaction between CK and gene sequences. The detailed mechanism about how CK regulates gene expression is still unknown.

**Figure 1:**
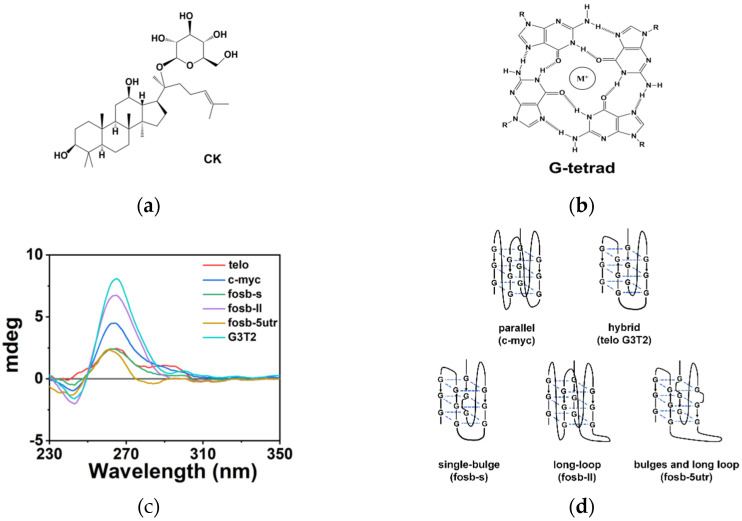
(**a**) The molecular formula of CK. (**b**) The illustration of G-tetrad. (**c**) The CD spectra of six G4 sequences in a buffer containing 40 mM Tris-HCl (pH 7.6), 8 mM MgCl_2_, and 60 mM KCl. (**d**) The schematic of expected structures of G4s used in this work.

The secondary structures of DNAs are important for their function. Most genes have non-canonical DNA structures that regulate their expression, such as triplex and quadruplex. G-quadruplex (G4) is a typical non-B DNA structure formed in a G-rich sequence [10], which widely exists in the human genome [11]. Recent research revealed that they play crucial roles in gene expression and regulation, including replication, transcription, and translation [12,13,14]. Interestingly, G4s were found to be significantly associated with oncogenes, and became potential targets for cancer intervention [15,16]. For example, *C-MYC* transcription was specifically downregulated by the stabilization of G4 in the *C-MYC* region, which further inhibited the development of triple-negative breast cancer [17]. The regulation of G4 has been expected as a new anticancer strategy, and many artificial ligands and natural products that interact with G4s are applied in the visualization of G4s and the inhibition of oncogenes [18,19]. Moreover, the stability of G4 was found to be greatly dependent on the surrounding environment. In cells, the presence of small components, proteins, polysaccharides, and metabolites results in molecular crowding conditions [20,21,22]. This significantly changed the stability of DNA structures, and should also be considered as an important factor in the regulation of gene expression. As CK has a structure of triterpenoid saponin (Figure 1a) [23,24,25], it might interact with DNA structures such as G4s through hydrogen bondings, change their stability, and regulate their expression. Discovering the effect of CK on G4 formation and stability will provide a new research direction for the pharmacological study of CK in the context of replication and transcription.

In this study, the effects of CK on G4s were investigated in detail. As a result, CK was demonstrated to stabilize G4s and regulate the structural equilibrium between the G4-complementary strand and full-matched duplex. Furthermore, the transcription of G4-containing templates was observed in the absence and presence of CK, and the results suggest that CK decreased the full-length transcripts and downregulated the expression of G4-containing genes. Our study is expected to provide a new perspective for DNA/RNA structural regulation by natural products and metabolites.

## 2. Results

### 2.1. G4 Characterization

G4 is commonly composed of more than two stacking G-tetrads, in which four guanines interact with each other through Hoogsteen base pairings (Figure 1b). Along with the π-π stacking between G-tetrads, the hydration changes also greatly affect the folded structures of G4s [26], which are usually dependent on the surrounding conditions. Moreover, the presence of single bulge and long loops in non-canonical G4s might accelerate the interaction between nucleotides and other molecules such as CK in the solution environment during the folding of G4. Therefore, as listed in Table 1, six different G4-forming sequences were selected to evaluate the effect of CK on G4s, including human telomere sequence (telo), G4 in oncogene *C-MYC* (c-myc), three non-canonical G4s with single bulge or long loops in oncogene *FOSB* (fosb-s, fosb-ll, fosb-5utr), and a normal G4 unrelated to disease (G3T2). In addition, a linear strand of 30 bases (linear) was also designed as a reference (Supplementary Materials Table S1).

The structures of six G4-forming sequences were firstly characterized by circular dichroism (CD) spectra. As shown in Figure 1c, c-myc and fosb-ll present parallel G4 structures, with positive peaks at 265 nm and negative peaks at 240 nm. On the other hand, telo, fosb-s, fosb-5utr, and G3T2 showed hybrid G4 structures, with positive peaks at 265 nm and 295 nm and negative peaks at 240 nm. Six G-sequences all formed G4 structures, and the schematic of the expected structures was drawn in Figure 1d.

### 2.2. Stabilization of G4s by CK

To characterize the effect of CK on G4 structures, the CD spectra were recorded after the addition of 100 μM CK (Figure S1). The results showed that the position of each peak had no change, while the intensity enhanced, suggested that the presence of CK stabilized all G4s and did not change their folding topology.

The change of melting temperatures (*T*_m_s) of G4s in the absence and presence of CK were then investigated at 260 nm by CD. Figure S2 shows the melting curves of six G4s in the absence and presence of CK, and the calculated *T*_m_s are listed in Table S2. According to the melting curves, all G4s were stabilized by the presence of CK. The Δ*T*_m_s of telo and G3T2 were +4.69 and +4.04 °C, respectively. The *T*_m_s of c-myc and fosb-ll were not calculated because *T*_m_s were too high to be detected. The *T*_m_s of fosb-s and fosb-5utr were not detected because they were not stable enough for the test. The melting experiments confirmed that the presence of CK stabilized G4s.

**Figure 2:**
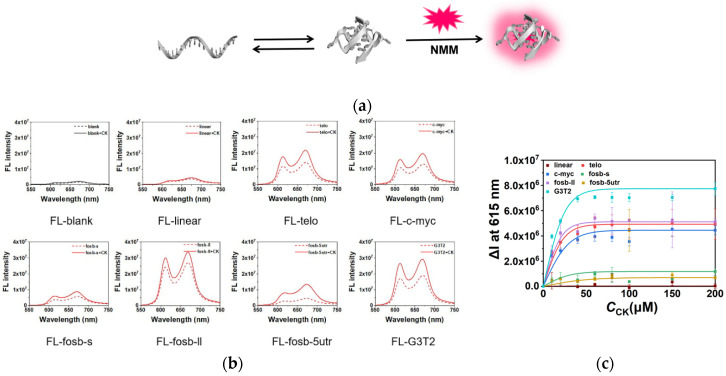
(**a**) The schematic demonstration of the equilibrium of coil DNA and G4 in the presence of fluorescence probe NMM. (**b**) The fluorescence spectra of NMM with different sequences in the absence (dash lines) and presence (solid lines) of 100 μM CK. (**c**) The fluorescence intensity changes (ΔI) at 615 nm in the presence of different concentrations of CK in the buffer containing 40 mM Tris-HCl (pH 7.6), 8 mM MgCl_2_, and 10 mM KCl.

The stabilization of G4s by CK were further analyzed by fluorescence. N-methyl mesoporphyrin IX (NMM), a common fluorescent ligand for G4, was utilized to quantify G4s in the solution. Coil DNA and G4 structure kept an equilibrium in the solution. In the presence of NMM, G4s combined with NMM and enhanced the fluorescence as shown in Figure 2a. The effect of CK on G4s is shown in Figure 2b. The presence of 100 μM CK had almost no influence on the fluorescence intensity of NMM, and only slight enhancement was observed in the presence of the linear sequence. Interestingly, the addition of CK obviously enhanced the fluorescence intensity in the presence of G4 sequences, indicating that the quantity of G4s was increased due to the stabilization of G4s by CK. Figure 2c shows the relationship between fluorescence changes and the concentrations of CK. The increment of fluorescence in the absence and presence of CK was expressed by ΔI at 615 nm, and the background fluorescence of NMM was subtracted. Consistent with previous results, all G4 sequences presented growing fluorescence intensities with the increase of CK concentration and gradually saturated at 60–100 μM of CK, which was dependent on the detailed sequences. On the contrary, the fluorescence of the linear sequence had no obvious enhancement, demonstrating that the enhancement of fluorescence was not generated from the interaction between NMM and CK. These results reveal that CK could stabilize G4s and promote the folding of G4s in the equilibrium between coil single strands and G4s. No competition between NMM and CK was observed, suggesting that the interaction between G4s and CK was different from the strong binding of NMM and G-tetrads.

The increment of fluorescence in the conditions of 60 mM and 100 mM K^+^ were also recorded for comparison (Figure S3), as potassium ion was reported to stabilize G4s [27]. Similar results were observed both in 60 mM and 100 mM K^+^. Stable G4s such as c-myc and G3T2 presented less of an increment in the high K^+^ condition due to the higher initial G4 quantity, while unstable G4s such as fosb-s and fosb-5utr showed an enhanced increment due to the stabilization of G4s by K^+^. The results confirm that the stabilization of G4s by CK was independent of K^+^. Based on these results, we speculated that unlike G4 ligands or K^+^, the interaction between CK and G4 was relatively weak. CK might work like crowding agent, interacting with G4s through hydrogen bondings with nucleotides and water molecules.

Moreover, the relationship between the stability changes of G4 and the surrounding environment was investigated in 10 wt% of each crowding agent by fluorescence melting curves. Utilizing glycerol, ethanol, ethylene glycol (EG), and polyethylene glycol 200 (PEG200) as crowding agents, the Δ*T*_m_s of telo were calculated and plotted with molecular weights, viscosities, and dielectric constants (Figure S4 and Table S3). The results revealed that the interactions between G4s and CK were complicated, and multiple factors worked together. Among them, the most related factor was the dielectric constant, suggesting that electrostatic interactions are important for the interactions between G4s and CK.

### 2.3. CK Facilitated G4 Folding in the Competition with Duplex

**Figure 3:**
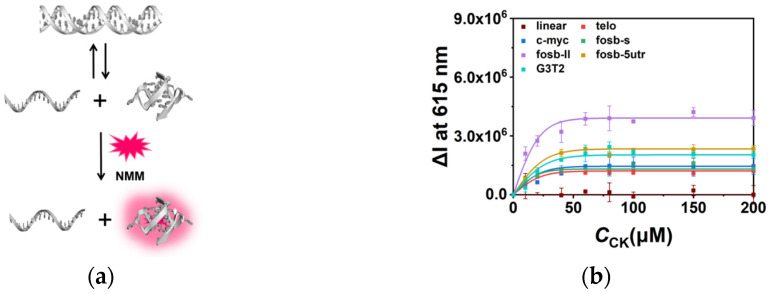
(**a**) The schematic demonstration of the equilibrium of duplex and G4-complementary sequence in the presence of fluorescence probe NMM. (**b**) The fluorescence intensity changes (ΔI) at 615 nm of NMM in the presence of different concentrations of CK in a buffer containing 40 mM Tris-HCl (pH 7.6) and 8 mM MgCl_2_ in the conditions with 10 mM K^+^.

The regulation of G4s by CK was also demonstrated in the G4-containing double strand system by fluorescence. The sequences telo, c-myc, fosb-s, fosb-ll, fosb-5utr, and G3T2 were observed with their complementary sequences, respectively (Table S1). The ratio of G4 and its complementary sequence was 1:1 to make an equilibrium between the G4 single strand and duplex (Figure 3a). NMM was utilized to quantify G4s. In the 10 mM K^+^ condition, all G4s presented a relatively poor stability compared with that of the full-matched double strands; therefore, weak initial fluorescence intensities were observed. In the presence of CK, as shown in Figure 3b, similar to the results of single strands, the fluorescence increased with the concentration of CK. Interestingly, in the conditions of 60mM and 100 mM K^+^ (Figure S5), the presence of CK induced a distinguishable enhancement of fluorescence in fosb-ll and G3T2 due to their higher stabilities in the competition with duplex. On the other hand, telo, c-myc, fosb-s, and fosb-5utr showed relatively low ΔI at 615 nm in 60 and 100 mM K^+^ due to their higher initial intensity or potential topology change, but the increasing tendency of fluorescence could be still observed in the presence of CK. These results confirm that CK assisted G4 folding in the competition between the G4-complementary single strand and full-matched duplex.

**Figure 4:**
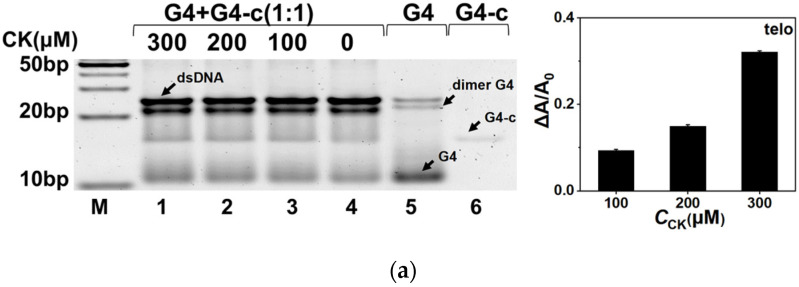

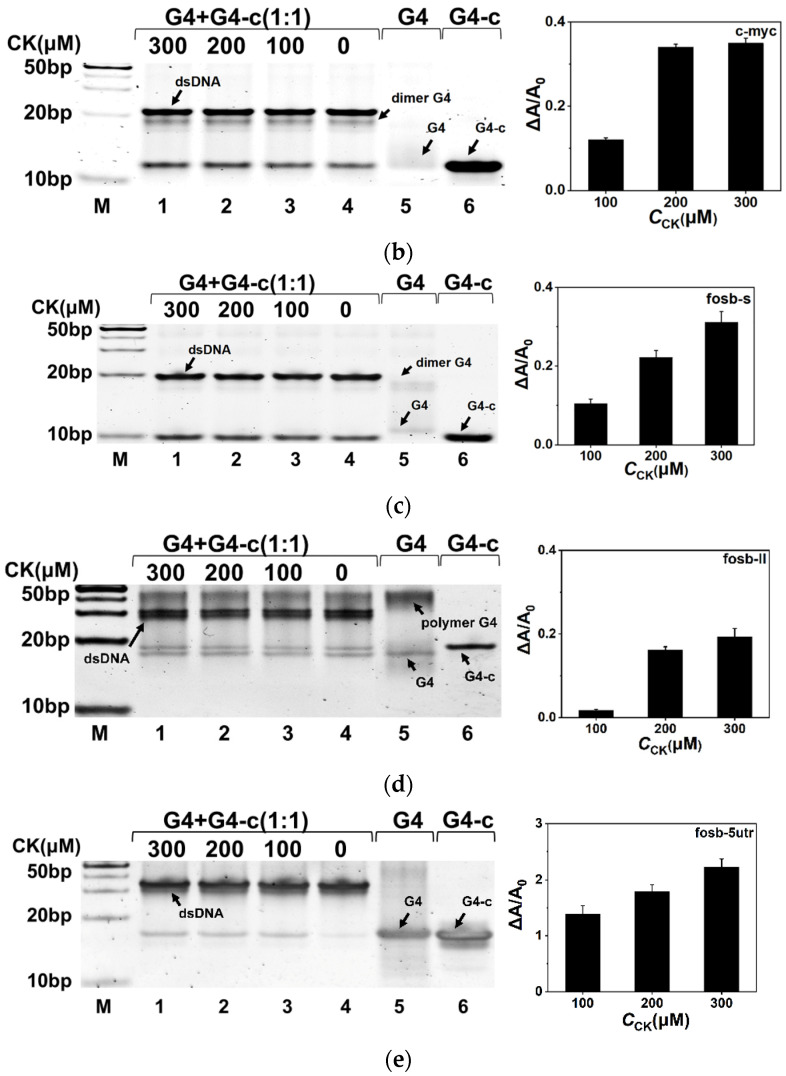
Native gel electrophoresis of (**a**) telo, (**b**) c-myc, (**c**) fosb-s, (**d**) fosb-ll, and (**e**) fosb-5utr. Lane M showed 10 bp marker. Lanes 1–4 were double strands of each G4 in the presence of 300, 200, 100, and 0 μM CK. Lane 5 was single strand of G4, and lane 6 was its complementary strand. The histograms on the right show the area changes of G4s in the presence of CK.

Gel electrophoresis was also performed to investigate the effect of CK on G4s in the competition with the full-matched duplex. DNA samples were first annealed in the buffer containing 100 mM K^+^, 20% PEG200, and 0, 100, 200, and 300 μM of CK, respectively. Then, the electrophoresis was conducted in 1× TBE buffer with 10 mM K^+^. The results are shown in Figure 4. G4 and G4-c stood for the corresponding G4 single strand and its complementary C-rich strand as the control. Most of the G4s were folded into intramolecular and intermolecular structures. The histograms on the right of Figure 4 show the area changes of G4s in the presence of CK, which were calculated from the detailed bands in each gel. For fosb-5utr, the bands of G4 were calculated for the G4 changes, while for c-myc and fosb-s, the bands of G4-c were used for calculation due to the poor dyeing effect of G4s. In addition, for telo and fosb-ll, both G4 and dimer G4/polymer G4 were calculated. As a result, an increased quantity of G4 or G4-c after the addition of CK was observed. However, no similar phenomenon was observed in G3T2 as shown in Figure S6, because the wide band of G3T2 was not conducive to quantitative analysis. These electrophoresis results demonstrate that CK facilitated G4 folding in the competition with duplex formation, corresponding to our fluorescence results.

### 2.4. Quantitative Characterization of the Effect on G4 by CK in the Duplex System

**Figure 5:**
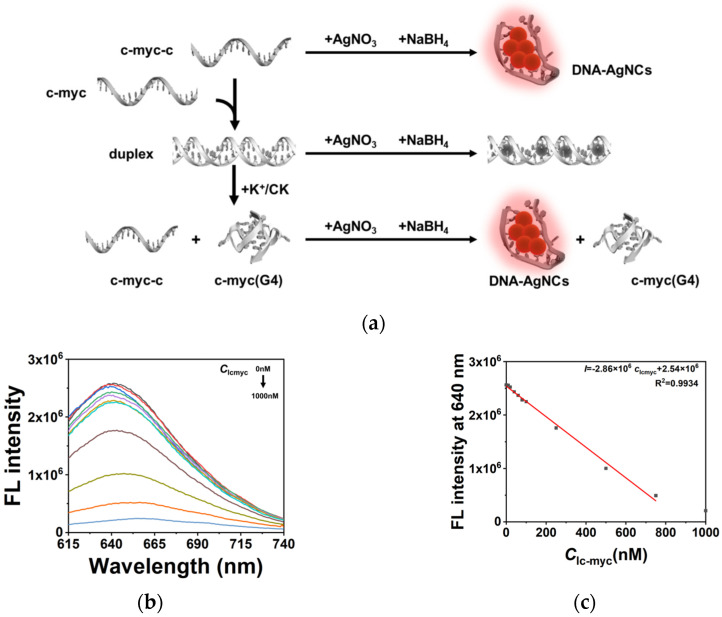

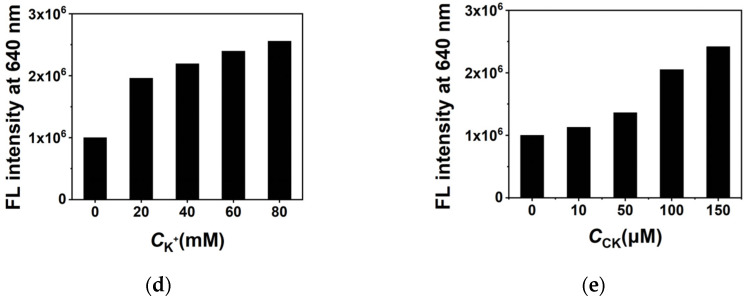
(**a**) The schematic demonstration of DNA-AgNCs synthesis and the sensing system for G4s. (**b**) The fluorescence spectra of 1000 nM c-myc-c DNA-AgNCs in the presence of different concentration of c-myc. (**c**) The linear relationship between target concentration and fluorescence intensity. (**d**) The histogram of fluorescence intensities in different concentrations of K^+^. (**e**) The histogram of fluorescence intensities in different concentrations of CK.

To quantitatively characterize the regulation of G4s by CK without any other ligands, DNA-templated silver nanoclusters (DNA-AgNCs) were designed as the probe for G4s. As shown in Figure 5a, the complementary sequence of c-myc (c-myc-c) was utilized as the template for fluorescent DNA-AgNCs. The synthesis of DNA-AgNCs was based on previous work with DNA:Ag^+^:NaBH_4_ at a ratio of 1:6:6 [28,29]. In the presence of c-myc, the formation of a duplex decreased the quantity of template c-myc-c and quenched the fluorescence, while the folding of the G4 structure in c-myc prevented the formation of a duplex and recovered the fluorescence. Based on the mechanism, a sensing method was built for the quantitative analysis of G4 folding. In the absence of K^+^, the folding of G4 was greatly inhibited, and c-myc presented as a linear structure and formed a duplex with c-myc-c. A linear range of linear c-myc between 10 nM and 750 nM was obtained with a detection limit of 10 nM (Figure 5b,c). The relationship between the concentration of linear c-myc (*c*_lcmyc_) and fluorescence intensity could be described as *I* = −2.86 × 10^6^ *c*_lcmyc_ + 2.54 × 10^6^ (R^2^ = 0.9934). The sensing system was then applied to the characterization of the folded G4s. The concentration of folded G4s could be described as *c*_total_ − *c*_lcmyc_. The DNA-AgNCs were synthesized with c-myc:c-myc-c at a ratio of 0.5:1 to obtain a better sensitivity. For example, the effect of K^+^ on G4 folding was measured. The fluorescence of DNA-AgNCs greatly enhanced with the increase of K^+^ (Figure 5d). According to the calculated *c*_lcmyc_, in 20 mM K^+^, 60.0% of the c-myc folded into G4 and almost 100% of the c-myc folded into G4 in 80 mM K^+^. This demonstrates that our sensing method is suitable for the characterization of G4s in the competition with duplex. Then, the effect of CK on G4 was quantitatively analyzed. Figure 5e shows the increased fluorescence intensity of DNA-AgNCs in the presence of 10, 50, 100 and 150 μM of CK, respectively. Based on the linear relationship in Figure 5c, the calculated *c*_lcmyc_ were 492, 411, 170, and 43 nM, respectively. As the total concentration of c-myc *c*_total_ was 500 nM, 91.4% of c-myc was folded into G4 in the presence of 150 μM of CK, confirming that CK significantly facilitated the folding of G4 in the competition with duplex formation, even in the absence of the G4 ligand and K^+^. To give an idea of the effect size of CK stabilizing G4s, the effect of strong G4 ligand pyridostatin (PDS) on G4 stabilization was measured for comparison (Figure S7) [30]. In the presence of 10 and 50 μM of PDS, the calculated *c*_lcmyc_ were 330 and 102 nM, respectively. As the total concentration of c-myc *c*_total_ was 500 nM, 79.6% of the c-myc was folded into G4 in the presence of 50 μM of PDS, which was even higher than the results of 100 μM of CK. These results demonstrate that the stabilization of G4 by CK was relatively weak compared to a strong G4 ligand such as PDS, corresponding to our fluorescence results with NMM.

### 2.5. The Regulation of CK in G-Quadruplex-Containing Transcription

**Figure 6:**
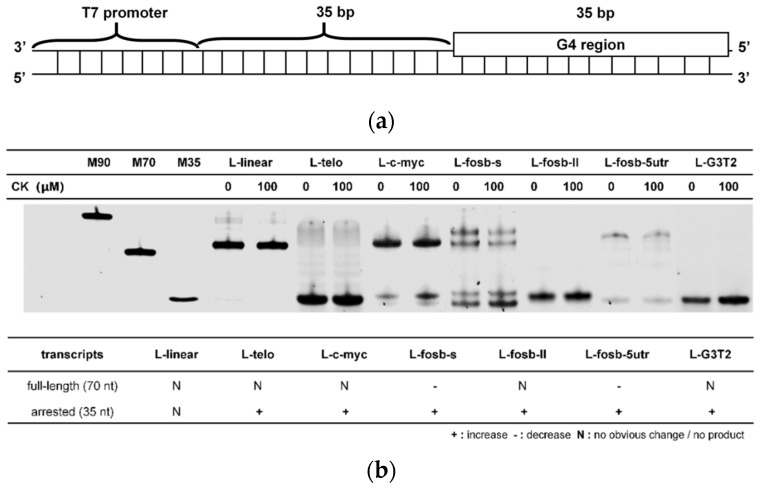

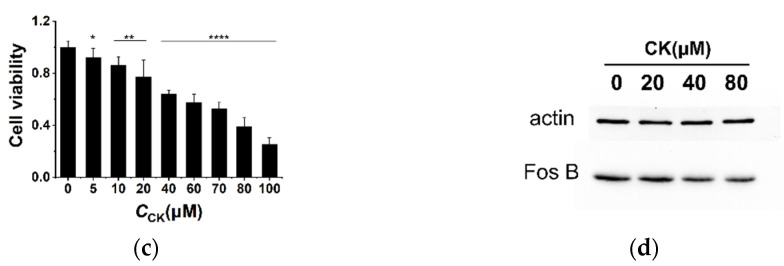
(**a**) The schematic illustrations of the DNA duplex template utilized for transcription. (**b**) The denatured gel electrophoresis of the transcripts of different templates in the absence and presence of CK. M90, M70, and M35 stand for linear markers with length of 90 nt, 70 nt, and 35 nt, respectively. The table below shows the variation trends of 35 nt and 70 nt transcripts in the presence of CK. (**c**) Cell viability of BEL-7402 cells after the treatment of 0, 5,10, 20, 40, 60, 70, 80, and 100 μM CK using CCK-8 assay. * *p* < 0.05, ** *p* < 0.01, **** *p* < 0.0001 vs. control. (**d**) Western blot analysis of FosB protein expression in BEL-7402 cells after the treatment of 0, 20, 40, and 80 μM CK for 24 h.

The effect of CK on G4 folding might further affect gene expression. Transcription is an important process in gene expression, and G4s were demonstrated to induce pause, slippage, and the arrest of transcription, playing crucial roles in expression regulation [31]. To analyze the effect of CK on G4s during transcription, six different G4 structures and a linear sequence were separately inserted to the template strand at a site 35 bases downstream from the T7 promoter region to avoid the effects of the G-quadruplexes on initiation complexes between T7 polymerase and template DNAs (Figure 6a and Table S4). As shown in Figure S7, the addition of CK increased the fluorescence in six G4-containing templates, corresponding to our results which show that CK can stabilize G4 structures. The transcripts of all templates were then characterized by denaturing gel electrophoresis. Based on our previous report [31], the full-length transcripts were 70 nt, the arrested transcripts were 35 nt, and the slipped transcripts were 70/35 ± 10 nt. As shown in Figure 6b, CK had almost no influence on the transcripts of L-linear. Interestingly, CK presented obvious effects on the transcripts of G4-containing templates. In L-fosb-s and L-fosb-5utr, the reduction of full-length transcripts was observed. The transcripts of all G4-containing templates showed the increase of arrested products, indicating that the stabilization of G4s by CK resulted in an arrest in transcription, interfering with the normal gene expression and decreasing the transcription efficiency.

To further confirm the effect of CK on the expression of G4-containing oncogenes, the expression of the *FOSB* gene was observed. *FOSB* is one proto-oncogene in the *FOS* gene family, which encodes FOS proteins that are implicated as regulators of cell proliferation, differentiation, and transformation. The sequences fosb-s, fosb-ll, and fosb-5tur were all potential G4-forming sequences in the *FOSB* gene. As shown in Figure 6c, CCK-8 analysis showed that the survival rate of BEL-7402 cells gradually decreased with the increase of CK concentration after 24 h of treatment with CK. In the subsequent Western blot experiments, 20, 40, and 80 μM were selected as the optimal concentrations of CK. The gene expression levels of the FosB protein in BEL-7402 cells were further measured by Western blot analysis. As shown in Figure 6d, the expression levels of the FosB protein were significantly decreased after the treatment with different concentrations of CK for 24 h compared to the control. The results correspond to the decrease of transcription efficiency in vitro. This provides new evidence that CK cannot only stabilize G4 structures, but can also participate in the regulation of G4-containing gene expression.

## 3. Discussion

CK, as the main metabolite of protopanaxadiol ginsenosides, shows excellent anticancer bioactivity by regulating the expression of key proteins [32,33,34,35]. However, little information is known about its detailed mechanism in gene regulation, especially about the potential interaction between CK and nucleic acids. The regulation of non-canonical DNA structures is one important way to regulate gene expression. G4, as a typical non-canonical structure, has been found to cause pause, slippage, and arrest in replication, transcription, and translation [31,36]. Therefore, the interaction between CK and G4 is significant for us to deeply understand the pharmacological effect of CK. Considering the structures of CK and G4, the interaction between CK and G4 would not be very strong as for most G4 ligands because of the lack of π–π stacking. Our experimental results also demonstrated that the saturation ratio of CK:G4 was over 40:1. The interaction between CK and G4 was complicated. Two possible explanations were considered. One is that CK has many saturated carbon structures, and these structures might interact with the π rings in G-tetrads through hydrogen bonding and CH–π interaction, as with crowding agents such as PEG [37], and result in the facilitation of G4 folding. Another one is that CK has many sugar residues, and they probably interact with the glycosides in nucleotides via hydrogen bonding. These two kinds of interaction might both exist, and our further investigation revealed that electrostatic interactions might be one of the most important factors in the interaction between G4s and CK.

To quantitatively describe the effect of CK on G4s, DNA-AgNCs were then utilized as the probe for the quantification of G4s. DNA-AgNCs have an excellent fluorescence property, which is greatly dependent on the structure of the template. A sensing method was designed based on the competition between duplex and G4 single strands. One of the G4 sequences in this work, c-myc, was selected as an example. This method can be used to detect linear c-myc in a range from 10 to 750 nM without other fluorescent ligands. Our method provided a general way to quantitatively evaluate the effects on G4 folding by various factors, such as K^+^. By calculating the concentration of linear c-myc and G4 c-myc, we found that 60.0% of the c-myc folded into G4 in 20 mM K^+^, while almost 100% of the c-myc folded into G4 in 80 mM K^+^. The same experiments were conducted with CK. Based on our experimental results, in the presence of 150 μM of CK, 91.4% of the c-myc was folded into G4, while in the presence of 10 μM of CK, only 1.6% of the c-myc had the structure of G4. Interestingly, the effect of CK at low concentrations was relatively weak, but high concentrations of CK greatly facilitated the folding of G4. The promotion of G4 folding was independent of K^+^ or other G4 ligands. The comparison of CK with the strong G4 ligand PDS was also observed by DNA-AgNCs. The results show that the presence of 50 μM PDS caused 79.6% of the c-myc to be folded into G4, while the presence of 50 μM CK only resulted in 17.8% of the c-myc being folded into G4. This demonstrates that the interaction between CK and G4 is much weaker than the interaction with G4 ligand PDS, which corresponds to our fluorescence and CD results.

The folding of G4s in genes would further greatly affect G4-containing transcription. For example, our previous work demonstrated that a temporary intermolecular triplex formation might unwind the G4 structure into its template strand and restart the transcription from arrest [31]. The stability change of G4 was crucial for G4 folding, especially in the competition with duplex. In our experiments, the presence of CK facilitated the folding of G4s in the competition with duplex, and further effects on transcriptions were observed. CK decreased the full-length transcripts and caused more slippage and arrest in transcription. The reduction of overall transcription efficiency by CK regulated the expression of oncogenes, explaining the anticancer mechanism of CK through DNA structural change. It was further confirmed by the decreased expression level of the FosB protein in BEL-7402 cells after the treatment of CK. The Western blot results corresponded to the transcription experiments in vitro, demonstrating that the downregulation of the FosB protein was caused by the G4 structural regulation. Our research is expected to provide a novel research field for the pharmacological study of CK.

## 4. Materials and Methods

### 4.1. Oligodeoxynucleotides and Materials

All DNA sequences used in this work were purchased from Shanghai Sangon Biotechnology Co., Ltd. (Shanghai, China) and used without further purification. Sequences are listed in Table S1. All DNA samples were dissolved in distilled water purified by a Thermo Type 2 system (Thermo, Langenselbold, Germany). The concentration of DNA was calculated from the absorbance at 260 nm.

BEL-7402 cells were purchased from the Institute of Biochemistry and Cell Biology, Academy of Science (Shanghai, China). RPMI Medium 1640 basic and fetal bovine serum (FBS) were purchased from Gibco (Grand Island, New York, NY, USA). Phosphate buffered solution (PBS), pancreatin, and penicillin-streptomycin solution were purchased from Hyclone (Logan, UT, USA). Anti-FosB and anti-β-actin were from Abcam (Cambridge, UK).

### 4.2. Circular Dichroism (CD) Measurement

CD spectra were recorded on a MOS-500 spectrophotometer (Bio-Logic, Seyssinet-Pariset, France). A total of 10 μM of DNA was dissolved in a buffer containing 40 mM of Tris-HCl (pH 7.6), 8 mM of MgCl_2_, and 60 mM of KCl in the absence and presence of 100 μM of CK. Samples were incubated at 90 °C for 5 min and then cooled to 4 °C over 2 h. The CD spectra were recorded in the range of 230 to 350 nm at a scan speed of 100 nm·min^−1^.

The CD melting experiments were conducted with a MOS-500 spectrophotometer (Bio-Logic, Seyssinet-Pariset, France). In the absence and presence of 100 μM of CK, 10 μM of DNA was dissolved in a buffer containing 40 mM of Tris-HCl (pH 7.6), 8 mM of MgCl_2_, and 10 mM of KCl. After incubation at 90 °C for 5 min, all samples were annealed from 90 °C to 15 °C for 2 h. The ellipticities at 260 nm were recorded when samples were heated from 15 °C to 95 °C at a rate of 1 °C·min^−1^.

### 4.3. Fluorescence Measurement

Fluorescence spectra were performed with a SpectraMax Paradigm Multi-Mode Detection Platform system (Molecular Devices, San Jose, CA, USA) at 37 °C. N-methyl mesoporphyrin IX (NMM) was utilized as the fluorescent ligand with a concentration of 3 μM. Then, 1.5 μM of DNA was dissolved in a buffer containing 40 mM of Tris-HCl (pH 7.6), 8 mM of MgCl_2_, and 10, 60, or 100 mM of KCl mixed with 0, 10, 20, 40, 60, 80, 100, 150, or 200 μM of CK at 4 °C. DNA solutions were incubated at 90 °C for 5 min and then cooled from 90 °C to 37 °C over 2 h. The fluorescence spectra of NMM from 550 nm to 750 nm were recorded with the excitation wavelength at 396 nm.

The melting temperature of telo in different molecular crowding conditions was measured with a QuantStudio 5 Real-Time PCR Instrument (Thermo, Woodlands, Singapore). A total of 1.5 μM of telo was dissolved in a buffer containing 40 mM of Tris-HCl (pH 7.6), 8 mM of MgCl_2_, 10 mM of KCl and 10 wt% of each cosolute. After incubation at 90 °C for 5 min, all samples were annealed from 90 °C to 4 °C for 2 h. The fluorescence intensity at 623 nm with the excitation wavelength at 470 nm was recorded when samples were heated from 4 °C to 90 °C at a rate of 0.9 °C·min^−1^.

### 4.4. Native Polyacrylamide Gel Electrophoresis

Native gel electrophoresis was conducted to determine the effect of CK on G4 DNA. In the absence and presence of 100, 200, and 300 μM of CK, G4s and complementary sequences with a ratio of 1:1 were mixed in a buffer containing 40 mM of Tris-HCl (pH 7.6), 8 mM of MgCl_2_, 100 mM of KCl, and 20% PEG200. Samples were incubated at 90 °C for 5 min and then cooled down to room temperature. The samples were mixed 1:1 with 2× loading buffer. Samples were conducted in 20% polyacrylamide gels containing 1× TBE buffer with 10 mM of KCl at 90 V for 2 h. After staining with SYBR Gold (Perkin Elmer Life Sciences, Waltham, MA, USA), scanning was performed with an iBright FL1000 Instrument (Thermo, Woodlands, Singapore).

### 4.5. Synthesis of DNA-AgNCs

DNA-AgNCs were synthesized according to a previous reference [28]. Briefly, 10 μM of DNA was mixed with 60 μM of AgNO_3_. After incubating at room temperature for 10 min, 60 μM of fresh NaBH_4_ was added with vigorous shaking for 5 min. The solution was kept in the dark at 4 °C for 24 h to form AgNCs. The fluorescence intensity of the samples after 10-fold dilution was measured by a SpectraMax Paradigm Multi-Mode Detection Platform system (Molecular Devices, CA, USA).

### 4.6. Transcription Assays

Transcription reactions were performed at 37 °C in a total volume of 50 μL. In the absence and presence of 100 μM CK, DNA templates (1.5 μM) were prepared in a buffer containing 40 mM of Tris-HCl (pH 7.6), 8 mM of MgCl_2_, 60 mM of KCl, and 3 μM of NMM overnight at 4 °C. Samples were incubated at 90 °C for 5 min and then cooled from 90 °C to 37 °C over 2 h. Then, 0.05% Tween-20, 4 mM of rNTP, 5 mM of dithiothreitol, and 1 U·μL^−1^ of recombinant RNase inhibitor were added. The fluorescence intensity of NMM at 615 nm was measured at 37 °C using a SpectraMax Paradigm Multi-Mode Detection Platform system (Molecular Devices, CA, USA). After adding 5 U·μL^−1^ of T7 RNA polymerase, all samples were incubated at 37 °C for 120 min of transcription. DNase I was utilized to digest the DNA templates and after 15 min incubation at 37 °C, the RNA transcripts were mixed with stop solution (80 wt% formamide, 10 mM of Na_2_EDTA, and 0.1% blue dextran) for further characterization. Samples were electrophoresed in a 10% polyacrylamide gel containing 7 M of urea in 1×TBE buffer at 120 V for 25 min. As the control, 35, 70, and 90 nucleotides of DNA sequences were electrophoresed. After staining with SYBR Gold (Perkin Elmer Life Sciences, Waltham, MA, USA), scanning was performed with an iBright FL1000 Instrument (Thermo, Woodlands, Singapore).

### 4.7. Cell Counting Kit-8 (CCK-8)

BEL-7402 cells in the logarithmic growth stage were digested with trypsin, centrifuged, resuspended, and counted by trypan blue staining. The cell suspension was diluted to a density of 5 × 10^3^ cells/well, inoculated into 96-well plates at 200 μL per well, and incubated at 37 °C in a 5% CO_2_ incubator. After 24 h of cell culture, drug treatment was started. For the control group (DMSO solvent) and CK group (5,10, 20, 40, 60, 70, 80, and 100 μM), six replicate wells were set up for each condition. After incubating for 24 h, 10 μL of CCK-8 solution was added to each well, and the absorbance value was measured at a wavelength of 450 nm using an enzyme marker after incubation for 30 min, and then cell viability was calculated.

### 4.8. Western Blot Analysis

After treatment with CK, the cells were harvested, washed twice with ice-cold PBS, and lysed with RIPA buffer on ice for 30 min. The lysates were centrifuged at 12,000 rpm for 30 min at 4 °C. To determine the protein concentration, a bicinchoninic acid protein assay was performed according to the manufacturer’s instructions. Equal amounts of protein were separated by electrophoresis on sodium dodecyl sulfate-polyacrylamide gels and transferred to a polyvinylidene fluoride membrane. The membrane was blocked with 5% skim milk in Tris-buffered saline containing Tween-20 for 1 h and then incubated with primary antibodies overnight at 4 °C. After three washes with the same buffer, the membranes were incubated with horseradish peroxidase-conjugated secondary antibodies. An enhanced chemiluminescence detection system was used to visualize the target proteins.

## 5. Conclusions

In conclusion, the stabilization of G4 structures by CK was demonstrated, and CK was found to regulate the equilibrium between duplex and G4-complementary sequences. Importantly, the presence of CK further affected the transcription of G4-containing templates, resulting in the pause and arrest of transcription, reduced full-length transcripts, and the downregulation of the expression level of related proteins. Thus, CK might be a potential anticancer drug targeted on the inhibition of oncogene expression.

## Figures and Tables

**Table 1:** The G4-forming sequences used in this work.

Name	Sequence (5′-3′)	Structure
telo	GGGTTAGGGTTAGGGTTAGGGTTA	Hybrid
c-myc	GGCCGCGGGCGGGGTTCGGG	Parallel
fosb-s	GGCGCGGGCGGGGCGCGGG	Hybrid
fosb-ll	GGGGCGGGTGACGTAAGCAGGGGGGCGGG	Parallel
fosb-5utr	GAGGTACAGCGGCATCCTGTGGGGGCCTGGG	Hybrid
G3T2	GGGTTGGGTGTGGGTTGGG	Hybrid

## Data Availability

Not applicable.

## Supplementary Materials

The following are available online. Table S1. Sequences used in this work; Table S2. The *T*_m_s of G4s in the absence and presence of 100 μM CK; Table S3. The *T*_m_s of telo in the absence and presence of CK in 10 wt% of crowding agents; Table S4. Transcription templates used in this work; Figure S1. CD spectra of (A) telo, (B) c-myc, (C) fosb-s, (D) fosb-ll, (E) fosb-5utr, (F) G3T2 in the absence and presence of 100 μΜ CK in a buffer containing 40 mM Tris-HCl (pH 7.6), 8 mM MgCl_2_ and 60 mM K^+^; Figure S2. The melting curves of (A) telo, (B) c-myc, (C) fosb-s, (D) fosb-ll, (E) fosb-5utr and (F) G3T2 in the absence and presence of 100 μΜ CK in a buffer containing 40 mM Tris-HCl (pH 7.6), 8 mM MgCl_2_ and 10 mM K^+^; Figure S3. The fluorescence intensity changes at 615 nm of NMM in the presence of different concentrations of CK in a buffer containing 40 mM Tris-HCl (pH 7.6) and 8 mM MgCl2 in the conditions with (A) 60 mM K^+^ and (B) 100 mM K^+^; Figure S4. The relationship between Δ*T*_m_s of telo in the absence and presence of CK and (A) viscosity, (B) molecular weight and (C) dielectric constant in 10 wt% of different crowding agents; Figure S5. The fluorescence intensity changes at 615 nm of G4s and their complementary sequences in the presence of different concentrations of CK in a buffer containing 40 mM Tris-HCl (pH 7.6), 8 mM MgCl_2_, in the conditions with (A) 60 mM K^+^ and (B) 100 mM K^+^; Figure S6. Native gel electrophoresis of G3T2. Lane M showed 10 bp marker. Lanes 1–4 were double strand of G3T2 in the presence of 300, 200, 100, and 0 μM CK. Lane 5 was single strand of G3T2, and lane 6 was the complementary strand of G3T2; Figure S7. The histogram of fluorescence intensities of DNA-AgNCs in different concentrations of pyridostatin (PDS); Figure S8. The fluorescence spectra of NMM in G4-containing templates (A) L-telo, (B) L-c-myc, (C) L-fosb-s, (D) L-fosb-ll, (E) L-fosb-5utr, (F) L-G3T2 in the absence and presence of 100 μΜ CK.

## Sample Availability

Samples of the compounds are not available from the authors.
