# Supplementary material for: Ginsenoside Compound K Assisted G-Quadruplex Folding and Regulated G-Quadruplex-Containing Transcription
Source: Molecules. 2021 Dec 3;26(23):7339. doi: 10.3390/molecules26237339 (PMC8659241; doi:10.3390/molecules26237339)
Supplement: Supplementary file 1 [file molecules-26-07339-s001.zip › molecules-1457599-supplementary.pdf]

Supporting Information

Ginsenoside compound K assisted G-quadruplex folding and regulated G-quadruplex-containing transcription

Yan Zhang, Zhidong Qiu, Ming Zhu and Ye Teng

School of Pharmacy, Changchun University of Chinese Medicine,

1035 Boshuo Road, Changchun 130117, China

Email: yteng28@163.com

Tables

Table S1. Sequences used in this work.

Name	Sequences (5’-3’)
telo	GGGTTAGGGTTAGGGTTAGGGTTA
c-myc	GGCCGCGGGCGGGGTTCGGG
fosb-s	GGCGCGGGCGGGGCGCGGG
fosb-ll	GGGGCGGGTGACGTAAGCAGGGGGGCGGG
fosb-5utr	GAGGTACAGCGGCATCCTGTGGGGGCCTGGG
G3T2	GGGTTGGGTGTGGGTTGGG
linear	ATTTCATAACTCAACCACGACACCATCGTG
telo-c	TAACCCTAACCCTAACCCTAACCC
c-myc-c	CCCGAACCCCGCCCGCGGCC
fosb-s-c	CCCGCGCCCCGCCCGCGCC
fosb-ll-c	CCCGCCCCCCTGCTTACGTCACCCGCCCC
fosb-5utr-c	CCCAGGCCCCCACAGGATGCCGCTGTACCTC
G3T2-c	CCCAACCCACACCCAACCC
linear-c	CACGATGGTGTCGTGGTTGAGTTATGAAAT

Table S2. The T_ms of G4s in the absence and presence of 100 μM CK.

**Sequence**	**T_m(°C)**	**T_m(°C)**	**ΔT_m(°C)**
	**-CK**	**+CK**	
telo	59.32	64.01	+4.69
c-myc	-[a]	-[a]	-
fosb-s	-[b]	-[b]	-
fosb-ll	-[a]	-[a]	-
fosb-5utr	-[b]	43.02	-
G3T2	75.23	79.27	+4.04

[a] Too high to be detected. [b] Not stable enough for the detection.

Table S3. The T_ms of telo in the absence and presence of CK in 10 wt% of crowding agents.

Sequence	Crowding agent	molecular weight ($g·mol^{-1}$)	viscosity (mPa·s)	dielectric constant (ε_r)	T_m(°C)		ΔT_m(°C)
					-CK	+CK	
telo	no cosolute	-	0.72	81.3	67.58	67.71	+0.13
	PEG200	200	1.54	67.7	68.63	70.07	+1.44
	ethanol	46	1.32	74.5	68.21	70.05	+1.84
	glycerol	92	1.64	77.9	69.40	69.82	+0.42
	EG	62	1.90	77.9	69.11	69.91	+0.80

Table S4. Transcription templates used in this work.

Name	Sequences (5'-3')
L-telo-a	GCCGTTTCGTAATTGGGATTGGGATTGGGATTGGGCAGAGAGAGCACCGAGCC TAGTTCGTGTCATCTCCTATAGTGAGTCGTATTAGTGATC
L-c-myc-a	GCCGTTTCGTAGTACGGGCTTGGGGCGGGCGCCGGCAGAGAGAGCACCGAGC CTAGTTCGTGTCATCTCCTATAGTGAGTCGTATTAGTGATC
L-fosb-s-a	GCCGTTTCGTAGTACTGGGCGCGGGGCGGGCGCGGCAGAGAGAGCACCGAGC CTAGTTCGTGTCATCTCCTATAGTGAGTCGTATTAGTGATC
L-fosb-ll-a	GCCGTTGGGCGGGGGGACGAATGCAGTGGGCGGGGCAGAGAGAGCACCGAG CCTAGTTCGTGTCATCTCCTATAGTGAGTCGTATTAGTGATC
L-fosb-5utr-a	GCCGGGGTCCGGGGGTGTCCTACGGCGACATGGAGCAGAGAGAGCACCGAGC CTAGTTCGTGTCATCTCCTATAGTGAGTCGTATTAGTGATC
L-G3T2-a	GCCGTTTCGTAGTATTGGGTTGGGTGTGGGTTGGGCAGAGAGAGCACCGAGCC TAGTTCGTGTCATCTCCTATAGTGAGTCGTATTAGTGATC
L-linear-a	GCCGTTTCGTAGTATTTGGGTTGTACCTATCGAGGCAGAGAGAGCACCGAGCCT AGTTCGTGTCATCTCCTATAGTGAGTCGTATTAGTGATC
L-telo-s	GATCACTAATACGACTCACTATAGGAGATGACACGAACTAGGCTCGGTGCTCTC TCTGCCCAATCCCAATCCCAATCCCAATTACGAAACGGC
L-c-myc-s	GATCACTAATACGACTCACTATAGGAGATGACACGAACTAGGCTCGGTGCTCTC TCTGCCGGCGCCCGCCCCAAGCCCATACTACGAAACGGC
L-fosb-s-s	GATCACTAATACGACTCACTATAGGAGATGACACGAACTAGGCTCGGTGCTCTC TCTGCCGCGCCCGCCCCGCGCCCAGTACTACGAAACGGC
L-fosb-ll-s	GATCACTAATACGACTCACTATAGGAGATGACACGAACTAGGCTCGGTGCTCTC TCTGCCCCGCCCACTGCATTCGTCCCCCCGCCCAACGGC
L-fosb-5utr-s	GATCACTAATACGACTCACTATAGGAGATGACACGAACTAGGCTCGGTGCTCTC TCTGCTCCATGTCGCCGTAGGACACCCCCGGACCCCGGC
L-G3T2-s	GATCACTAATACGACTCACTATAGGAGATGACACGAACTAGGCTCGGTGCTCTC TCTGCCCAACCCACACCCAACCCAATACTACGAAACGGC
L-linear-s	GATCACTAATACGACTCACTATAGGAGATGAGACGAACTAGGCTCGGTGCTCTC TCTGTCTCGATAGTTACAACCCAAATACTACGAACCGGC

Figures

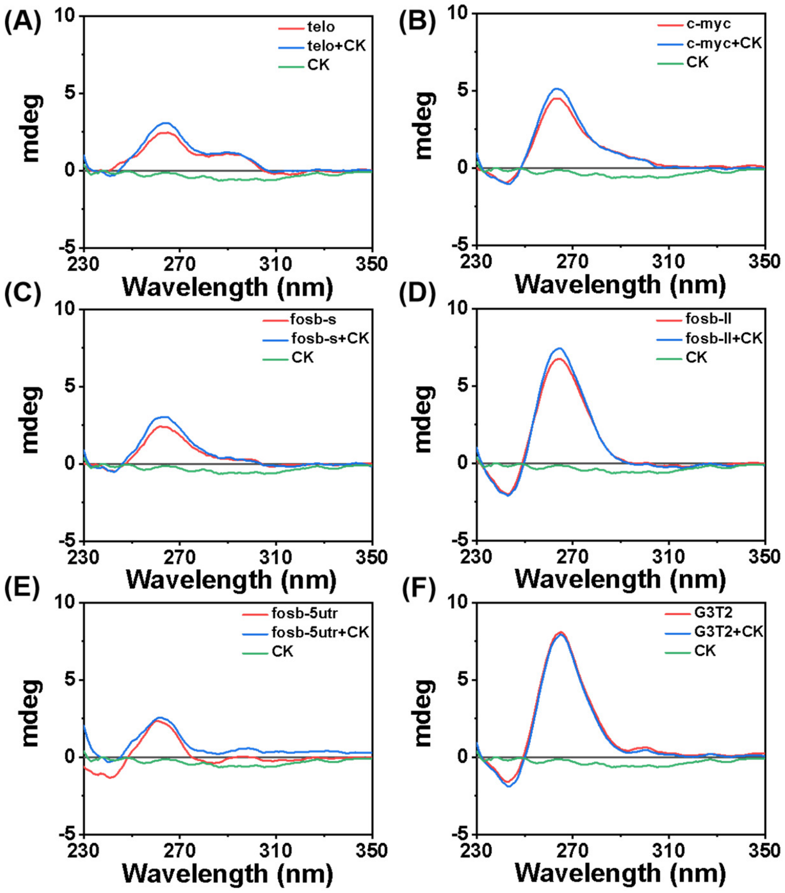

Figure S1. CD spectra of (A) telo, (B) c-myc, (C) fosb-s, (D) fosb-ll, (E) fosb-5utr, (F) G3T2 in the absence and presence of 100 μM CK in a buffer containing 40 mM Tris-HCl (pH 7.6), 8 mM $MgCl_2$ and 60 mM K^+.

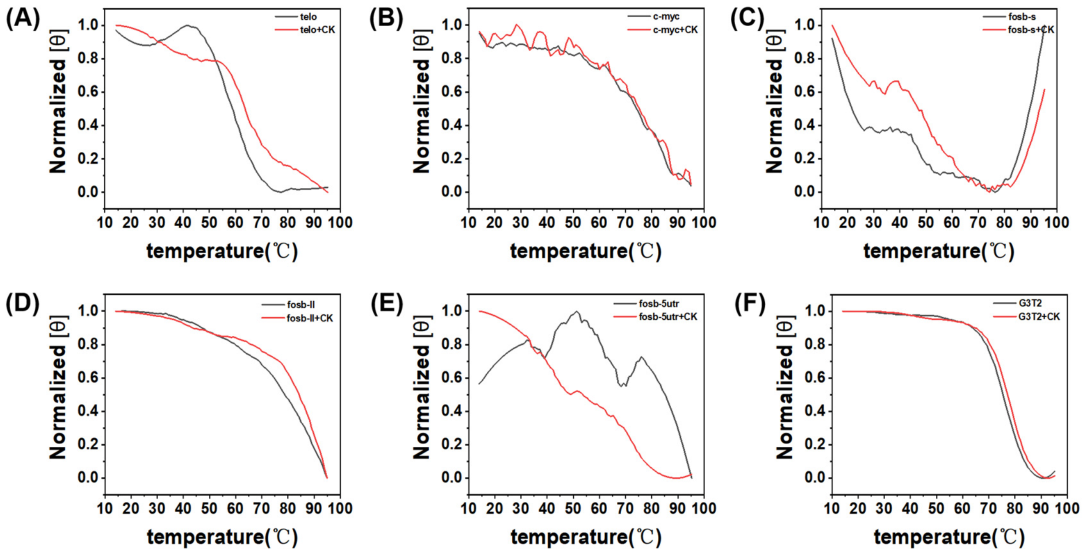

Figure S2. The melting curves of (A) telo, (B) c-myc, (C) fosb-s, (D) fosb-ll, (E) fosb-5utr and (F) G3T2 in the absence and presence of 100 μM CK in a buffer containing 40 mM Tris-HCl (pH 7.6), 8 mM $MgCl_2$ and 10 mM K^+.

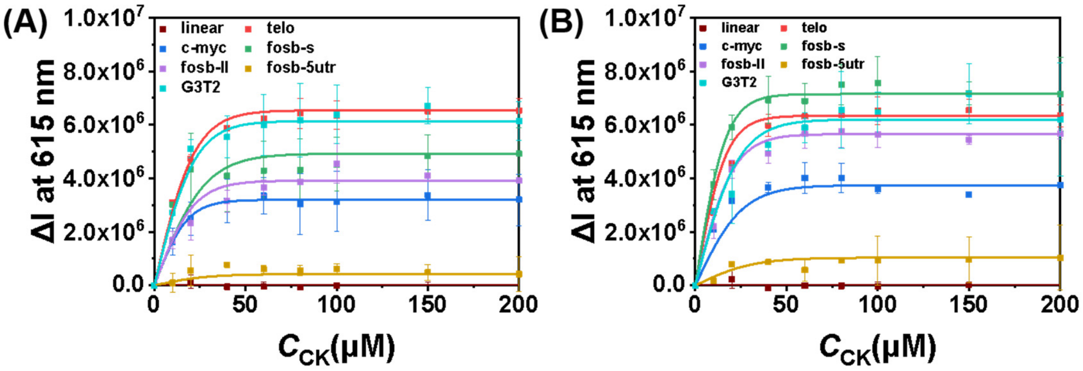

Figure S3. The fluorescence intensity changes at 615 nm of NMM in the presence of different concentrations of CK in a buffer containing 40 mM Tris-HCl (pH 7.6) and 8 mM $MgCl_2$ in the conditions with (A) 60 mM K^+ and (B) 100 mM K^+.

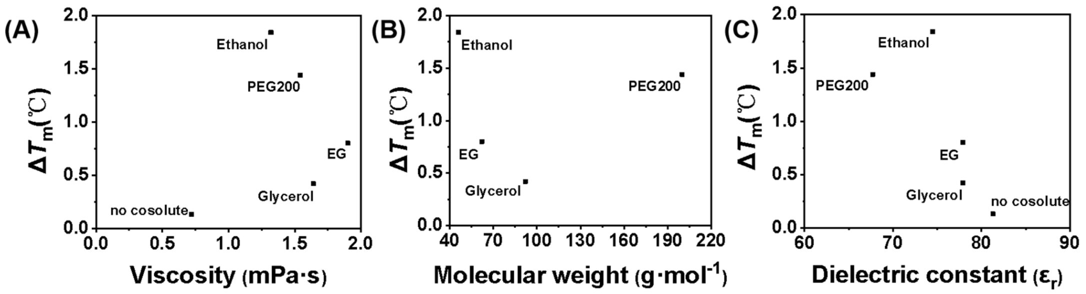

Figure S4. The relationship between ΔT_ms of telo in the absence and presence of CK and (A) viscosity, (B) molecular weight and (C) dielectric constant in 10 wt% of different crowding agents.

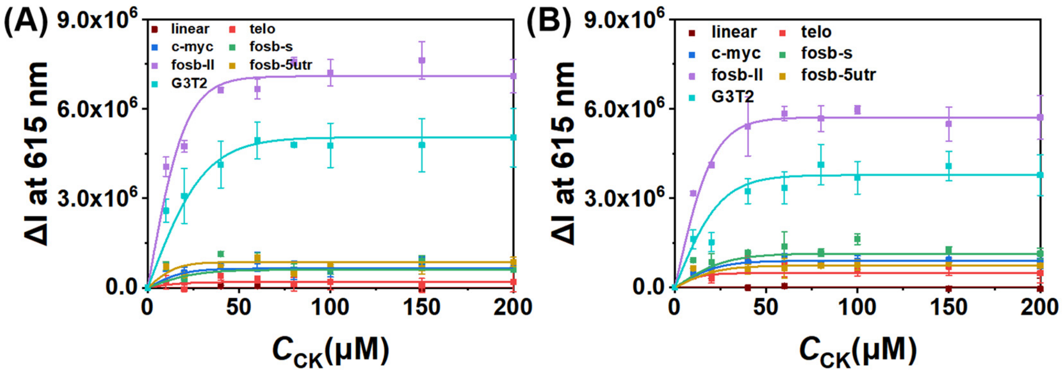

Figure S5. The fluorescence intensity changes at 615 nm of G4s and their complementary sequences in the presence of different concentrations of CK in a buffer containing 40 mM Tris-HCl (pH 7.6), 8 mM $MgCl_2$, in the conditions with (A) 60 mM K^+ and (B) 100 mM K^+.

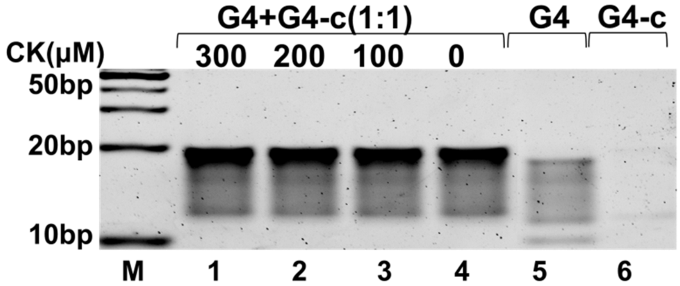

Figure S6. Native gel electrophoresis of G3T2**.** Lane M showed 10 bp marker. Lanes 1-4 were double strand of G3T2 in the presence of 300, 200, 100, and 0 μM CK. Lane 5 was single strand of G3T2, and lane 6 was the complementary strand of G3T2.

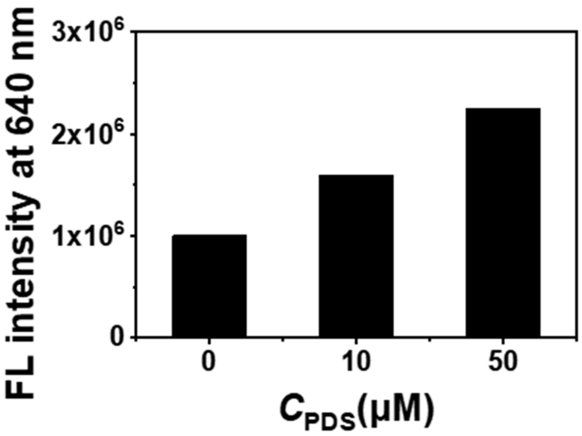

Figure S7. The histogram of fluorescence intensities of DNA-AgNCs in different concentrations of pyridostatin (PDS).

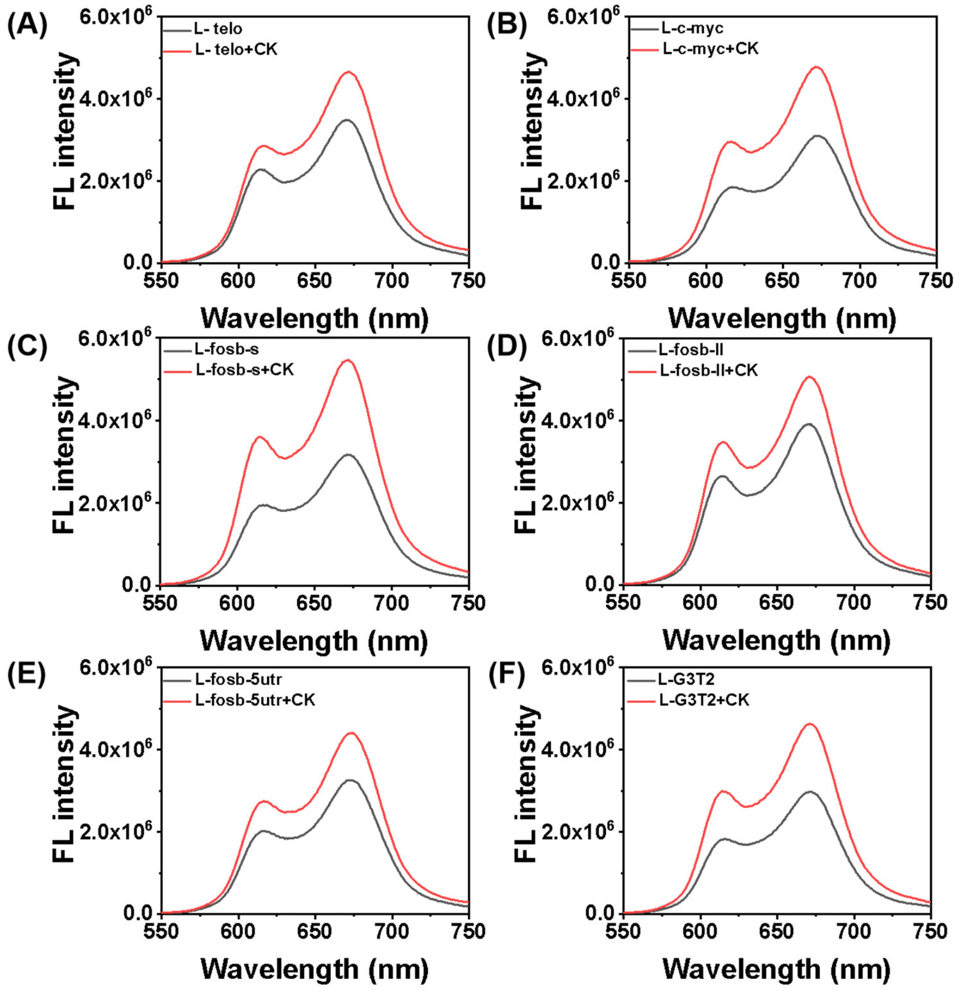

Figure S8. The fluorescence spectra of NMM in G4-containing templates (A) L-telo, (B) L-c-myc, (C) L-fosb-s, (D) L-fosb-ll, (E) L-fosb-5utr, (F) L-G3T2 in the absence and presence of 100 μM CK.
